# Relapsing Fever in Carlisle

**Published:** 1871-11

**Authors:** 


### Relapsing Fever in Carlisle.

Several of the cases of relapsing fever observed in Carlisle, it would appear, are very characteristic. Persons while engaged at business are suddenly seized with headache, pains in the epigastric region, back, and limbs, with rigors, and with severe vomiting, which obstinately continues for some days. A reaction shortly follows the chief symptoms, there being a hot, dry skin, great thirst, full quick pulse (which soon rises to from 120 to 150 per minute), little or no sleep for two or three days and nights, general restlessness, hurried suppressed breathing, face suffused and expression anxious, some feeling of prostration, impaired appetite, giddiness, and intolerance of light and sound. The skin assumes a bronzed hue, or jaundice may appear. Sweating, at first slight, becomes profuse. The tongue generally continues moist, although a white fur covers it; occasionally, however, a dry brown line runs along the centre. The bowels are confined. The urine is either suppressed or scanty and high-colored; and when allowed to rest for a short time, half or more of the entire volume may consist of deposit. There is enlargement of the liver. Delirium manifests itself during some part of the disease. The eyeballs are affected in some instances. Towards the end of a week from the commencement of the disease, as the symptoms mentioned greatly increase in severity, a crisis ensues; it may be by sweating which saturates the bedclothes around the patient, by a free flow of urine, and by several loose motions of the bowels. To the patient recovery seems so complete that the next day after the crisis he is up, and in the course of three or four days, without the permission of his medical man, may go out for a walk or resume business. At the close of an apyrectic week or so of happiness there is a *relapse* of all the former symptoms. This second attack is milder and of shorter duration than the former. A second apyrectic interval occurs, and a *second relapse*, which may be more grave than the primary attack. Those affected by the disease are the improperly and ill-fed people who live in dirty, crowded, badly ventilated houses.—*Lancet.*

